# Supplementary material for: Transcriptome Analysis Reveals Genes and Pathways Associated with Salt Tolerance during Seed Germination in Suaeda liaotungensis
Source: Int J Mol Sci. 2022 Oct 13;23(20):12229. doi: 10.3390/ijms232012229 (PMC9603052; doi:10.3390/ijms232012229)
Supplement: Supplementary file 1 [file ijms-23-12229-s001.zip › Supplementary Figures S1-S4.pdf]

International Journal of
Molecular Sciences

MDPI

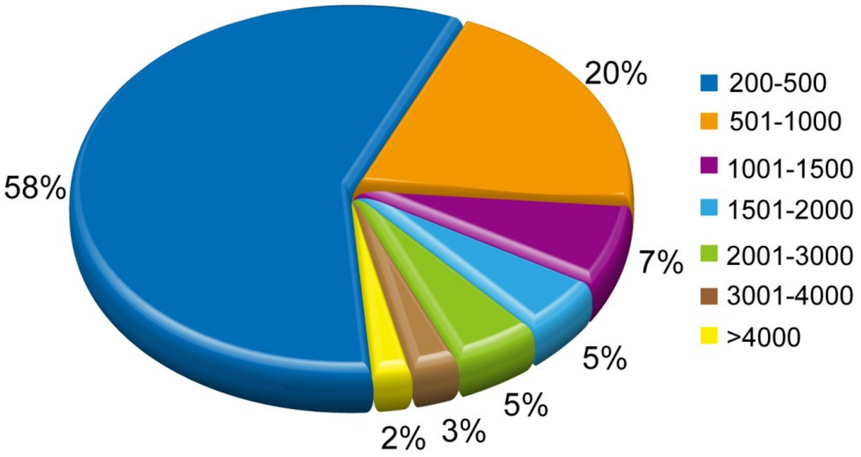

Figure S1. Length distribution of unigenes generated via *de novo* assembly. Different colors represent the percentage of unigenes in a certain length range, including 200–500 bp (dark blue), 501–1000 bp (organe), 1001–1500 bp (purple), 1501–2000 bp (light blue), 2001–3000 bp (green), 3001–4000 bp (dark coffee), and more than 4000 bp (yellow).

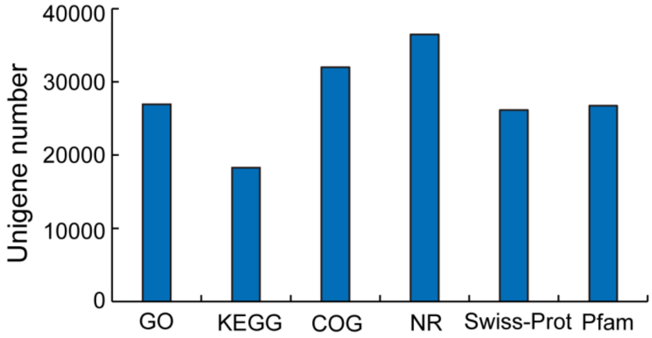

Figure S2. The distribution of unigenes annotated to six public databases. Six public databases including NCBI non redundant protein (NR), Clusters of Orthologous Groups (COG), Gene Ontology (GO), Kyoto Encyclopedia of Genes Genomes (KEGG), Protein Family (Pfam), and Swiss-Prot public protein database.

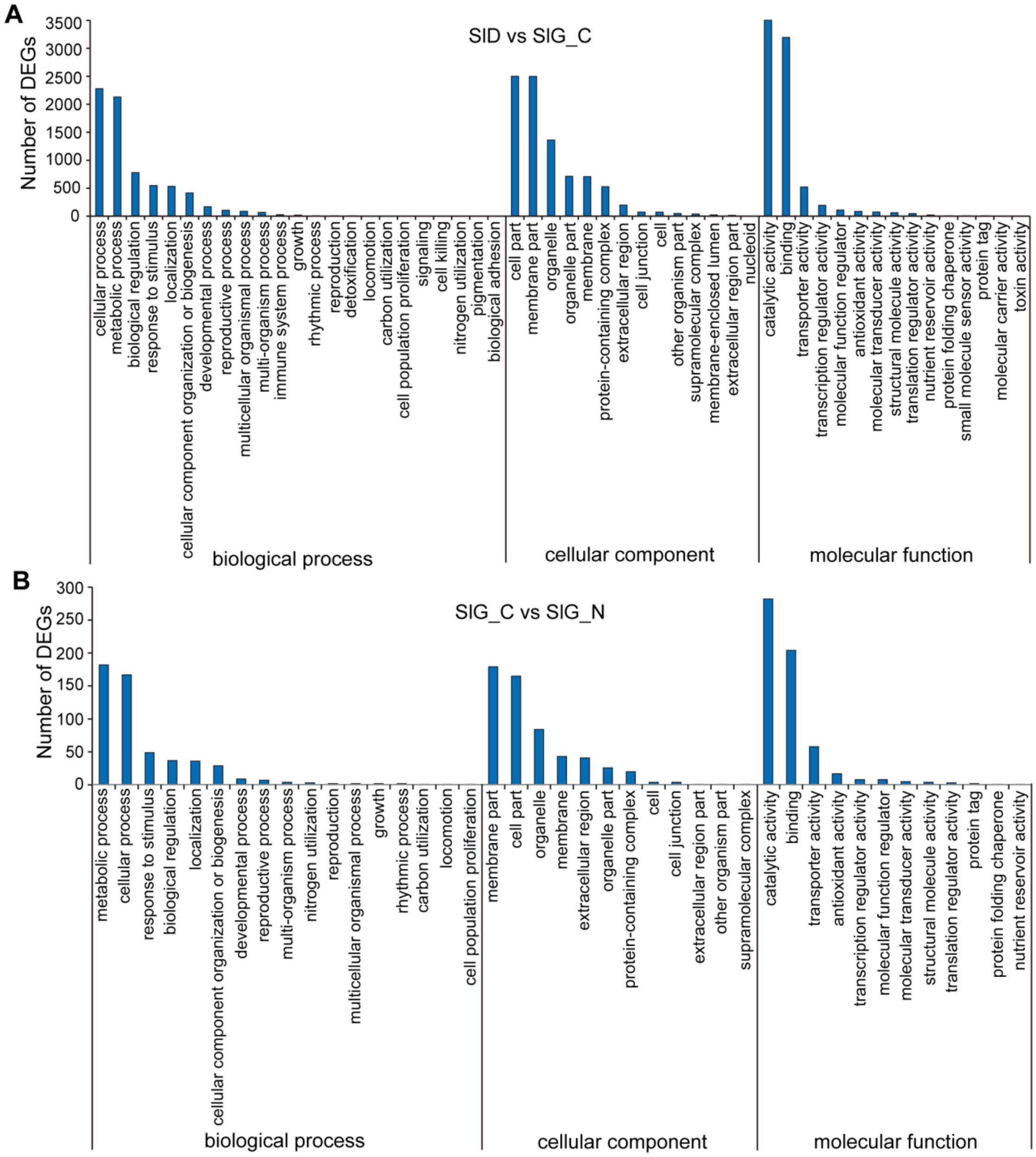

Figure S3. GO functional annotation analysis based on the differentially expressed genes in the SlD vs SlG_C (**A**) and SlG_C vs SlG_N (**B**) groups in term of biological process, cellular component and molecular function.

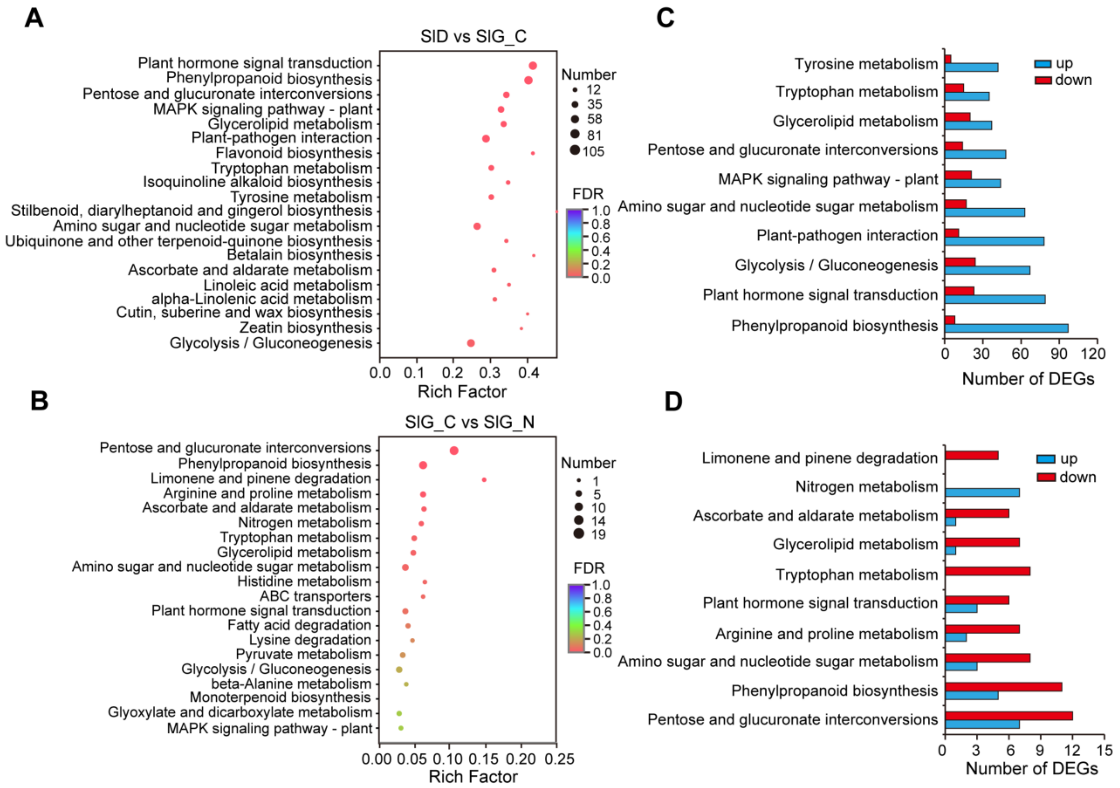

Figure S4. KEGG pathway enrichment analysis based on the DEGs in the SlD vs SlG_C (**A**) and SlG_C vs SlG_N (**B**) groups. The X-axis represents the enrichment factor, and the Y-axis represents the pathway name. Colors represent the FDR, and the size of the dot represents the number of DEGs. The top 10 pathways of DEGs enriched in the SlD vs SlG_C (**C**) and SlG_C vs SlG_N (**D**) groups. Up-regulated genes (blue) and down-regulated genes (red). P_{adj}-*value* <0.05.
